# Re: Prognosis of prostate cancer and prostate - specific antigen levels

**DOI:** 10.1590/S1677-5538.IBJU.2018.0867

**Published:** 2019-07-27

**Authors:** Beuy Joob, Viroj Wiwanitkit

**Affiliations:** 1 Sanitation 1 Medical Academic Center, Bangkok Thailand; 2 Honorary professor, Dr DY Patil University, Pune, India

*To the editor,*

We read the publication on “Prognosis of patients with prostate cancer and middle range prostate - specific antigen (PSA) levels of 20 – 100 ng / mL” with a great interest (1). Iwamoto et al. noted that *“PSA is a useful biomarker for predicting prognosis at levels between 20 and 70 ng / mL (1)”* and *“When the PSA level reaches approximately 70 ng / mL, prognosis might bottom and reach a plateau (1)”.* We would like to share ideas on this report. We agree that there might be a plateau phenomenon in case of high PSA level. Nevertheless, we should also be aware on the possible false negative result in patients with low PSA level (2). As an immunological test, the prozone effect is possible and this might lead to aberrant low PSA level (3). In that case, the prognosing might be affected.
